# A new approach for the quantification of synchrony of multivariate non-stationary psychophysiological variables during emotion eliciting stimuli

**DOI:** 10.3389/fpsyg.2014.01507

**Published:** 2015-01-20

**Authors:** Augustin Kelava, Michael Muma, Marlene Deja, Jack Y. Dagdagan, Abdelhak M. Zoubir

**Affiliations:** ^1^Hector Research Institute of Education Sciences and Psychology, Eberhard Karls Universität TübingenTübingen, Germany; ^2^Department of Electrical Engineering and Information Technology, Technische Universität DarmstadtDarmstadt, Germany; ^3^Department of Psychology, Technische Universität DarmstadtDarmstadt, Germany

**Keywords:** multivariate, synchrony, coherence, emotion regulation, psychophysiology, time series, frequency, latent variables

## Abstract

Emotion eliciting situations are accompanied by changes of multiple variables associated with subjective, physiological and behavioral responses. The quantification of the overall simultaneous synchrony of psychophysiological reactions plays a major role in emotion theories and has received increased attention in recent years. From a psychometric perspective, the reactions represent multivariate non-stationary intra-individual time series. In this paper, a new time-frequency based latent variable approach for the quantification of the synchrony of the responses is presented. The approach is applied to empirical data, collected during an emotion eliciting situation. The results are compared with a complementary inter-individual approach of Hsieh et al. (2011). Finally, the proposed approach is discussed in the context of emotion theories, and possible future applications and limitations are provided.

## Introduction

Researchers agree that emotion eliciting situations are accompanied by changes of multiple variables associated with subjective, physiological and behavioral responses. The reasons for a possible coupling of the response variables is a topic of ongoing discussion in various emotion theories. There is no uniform terminology to describe the simultaneous changes of the response variables. The term “coherence” is frequently used (e.g., Rosenberg and Ekman, 1994; Reisenzein, 2000; Mauss et al., 2005; Sze et al., 2010; Herring et al., 2011; Hsieh et al., 2011; Dan-Glauser and Gross, 2013) to describe the simultaneity of changes in the response variables. Further terms that are used in the research community to describe the interrelation of the responses are “synchronization,” “organization of response systems,” or “concordance,” Throughout this paper, the terms synchronization and synchrony are interchangeably used to describe the simultaneous changes of response variables. This article introduces a new approach for the quantification of the synchrony of the response variables that is able to account for non-stationarity. A signal is non-stationary if its mean and covariance function are time-varying (Brillinger, 2001). First, different assumptions regarding the functionality of the synchrony concept in different emotion theories are explained. Subsequently, the new approach, which basically consists of two steps, is introduced. In a first step, time-frequency based bivariate coherence measures are derived (Muma et al., 2010). In a second step, these measures are used in an state space modeling approach to obtain an overall synchrony measure of the simultaneous activation of psychophysiological responses. The approach is then applied to empirical data collected during an emotion eliciting situation and compared with a complementary approach of Hsieh et al. (2011). Finally, the proposed approach is discussed in the context of emotion theories, and possible future applications and limitations are provided.

### On the functionality of synchrony of responses in emotion theories

Emotion theories make different assumptions regarding the functionality of a synchrony of response variables: Basic emotion theories state that different emotions have distinct and coordinated patterns of physiological responses. According to basic emotion theories, the specific psychophysiological response variables are activated simultaneously during an emotional experience but are less associated with each other during rest (Tomkins, 1962; Izard, 1977; Ekman, 1992; Levenson, 1994). A synchronized response is desirable as it prepares the body for an adequate reaction to a stimulus and leads to an appropriate reaction to environmental demands (Tomkins, 1962; Izard, 1977; Ekman, 1992; Levenson, 1994). Different emotional responses are organized by central mechanisms in the brain, such as the amygdala (e.g., Whalen et al., 1998; LeDoux, 2000; Murphy et al., 2003), orbitofrontal cortex (e.g., Hornak, 2003; Murphy et al., 2003; Goodkind et al., 2012), the insular (e.g., MacLean, 1990; Damasio et al., 2000; Murphy et al., 2003), and other brain regions (e.g., Ekman, 1992; Panksepp, 2008; Izard et al., 2011). The synchronized specific responses in time and intensity have been interpreted as evidence for the existence of a causal mechanism (e.g., Kettunen et al., 1998; Levenson, 2003). Further, emotions can also activate so called ‘affect programs’ that include behavioral and physiological changes, that might be similar for different individuals (Tomkins, 1962; Ekman and Cordaro, 2011). For example, fear, anger, or amusement may be accompanied by a specific reaction pattern in terms of subjective experience, physiology, and behavior (Ekman, 1992; Murphy et al., 2003; Levenson, 2011; Panksepp and Watt, 2011).

A different viewpoint is taken by so-called dimensional approaches that do not classify the emotional experience in distinctive categories, such as anger or fear. Instead, these approaches discriminate between different emotional states by introducing a two-dimensional space that is spanned by valence (pleasure/displeasure) and arousal (activated/deactivated) as sufficient means to discriminate between different emotional states (Russell, 1980, 2003; Barrett and Russell, 1998; Barrett, 2006). Higher dimensional spaces have been proposed by e.g., Bradley and Lang (1994) or Fontaine et al. (2007). The assumption that an event causes an emotion and the emotion causes a synchronized, specific change in cognition, behavior, and physiological reaction is criticized by Russell (2003). Instead, in the conceptual framework of the core affect, the hypothesis is made that that emotions do not have a common cause. From this perspective, a synchrony between the responses is not required (see also Barrett, 2006; Russell, 2009). On grounds of genetic differences, personal experiences (e.g., Lykken and Tellegen, 1996), responsiveness to stimuli, attributions, and other factors, individuals can respond differently to the same emotion eliciting situation (Russell, 2003). Hormonal changes, endocrine dysfunction, illness, satiety and diurnal rhythms can internally influence the emotional response (Russell, 2003), which results in very specific patterns of psychophysiological variables on the intra- and inter-individual level.

A third body of research relies on the concept of appraisal. Appraisal indicates an evaluation of the situation regarding its personal significance (Lazarus, 1991) and therefore, in the same situation there can be a great variation between the emotional state of individuals (Scherer et al., 2006; Kuppens et al., 2009). Still, a synchronous response during an emotional episode is expected (Scherer, 2009). Some of the appraisal theories view the appraisal of a situation, and not the event itself (as projected by the basic emotion theory; Russell, 2003), as a causal mechanism responsible for the elicitation of an emotion (e.g., Schachter and Singer, 1962; LeDoux, 1989; Roseman et al., 1990; Scherer, 1993). Lewis (2005), on the other hand, assumes a recursive, complex relationship between subsystems of the nervous system and hence, not a linear relationship of one causal mechanism followed by a cascade of responses. Grandjean et al. (2008) consider various feedback loops between the synchronized response of the peripheral-, motivational-, monitor-, cognitive-, and motor-system to be responsible for the conscious awareness of an emotion (e.g., Scherer, 1984, 1987, 2009; Fries, 2005). The synchrony of multiple response variables, however, is considered to be necessary for a conscious emotional experience (Grandjean et al., 2008). Also, several appraisal approaches support discrete categories of emotion (e.g., Oatley and Johnson-laird, 1987; Roseman et al., 1990) while others approve the dimensional perspective (e.g., Scherer et al., 2006; Kuppens et al., 2012; Wu et al., 2012). The above approaches are theoretical and the question remains how to quantify the synchrony of the response variables. The specific response variables constitute multivariate time series with time-varying distributions. Additionally, (Dan-Glauser and Gross, 2013) recently stated that defining a measure of synchrony is a challenging and timely topic in emotion theory. In the following section, the results concerning the synchrony of peripheral physiological response variables during emotion eliciting situations are reviewed.

### The synchrony of peripheral physiological response variables during emotion eliciting situations: a brief review of results

In early psychological research of emotion and stress, the synchrony of peripheral physiological measures was a major focus of the analyses (Wenger, 1942; Lacey and Lacey, 1958; Lazarus et al., 1963; Nesse et al., 1985). For example, the autonomic response system (ANS) plays an important role during stress (e.g., Carroll et al., 2009; Bibbey et al., 2013), posttraumatic stress disorder (e.g., Zucker et al., 2009; Ehlers et al., 2010) or anxiety-disorders like panic-attacks (e.g., Roth et al., 1988; Meuret et al., 2011). Information on the temporal interdependences of peripheral physiological measures can enhance the understanding of the underlying functioning of the ANS (Kettunen et al., 1998; McAssey et al., 2013) and can provide important information about the psychophysiological processes during an emotional episode (McAssey et al., 2013). However, it is unclear under which conditions, and to what exact quantitative extent, a synchronous physiological reaction occurs (Hsieh et al., 2011; McAssey et al., 2013). Applying intra-individual time series models, (Kettunen et al., 1998) reported that the synchronization between electrodermal activity and heart rate within an individual is associated with a higher level of arousal and behavioral activity (see also Lazarus et al., 1963). In their stochastic network configuration approach, (Hsieh et al., 2011) found three clusters within an overall system that is formed by 15 psychophysiological signals: one behavioral cluster and two physiological clusters (blood pressure and cardiovascular parameters). They reported a higher synchronization between the different clusters as the intensity of an emotional stimuli was measured from a neutral condition, suggesting that there is a higher association during an emotional experience episode. A higher synchrony was also reported within each cluster. Based on brain activity analysis, (Costa et al., 2006) found by using a synchronization index, a higher synchrony of various EEG channels during emotional film stimuli than during neutral film clips. Their results indicate a higher information exchange during emotional responses (for similar results see also e.g., Miskovic and Schmidt, 2010).

Numerous studies on psychophysiological correlates of emotional stimuli have been undertaken. However, reactions from emotion eliciting stimuli are not universal on the inter- and intra-individual levels. Individuals vary in the intensity and duration of an emotional episode in terms of subjective experience, physiological and behavioral reactions (e.g., Grandjean et al., 2008; Kuppens et al., 2009). Thus, physiological response patterns during emotion tend to differ between individuals (e.g., Marwitz and Stemmler, 1998; Kristjansson et al., 2007). Kristjansson et al. (2007) applied a two level growth curve model, in which the first level explains the variance within participants and the second level the variance between participants. Marwitz and Stemmler (1998) used correlations and ANOVA to analyze individual response specificity. The physiological responses were found to be influenced by the individual appraisal (see Scherer, 2009, for an overview), emotion regulation (e.g., Gross and Levenson, 1993, 1997; Dan-Glauser and Gross, 2011), and context specific attributes (see Cacioppo et al., 1992, for an overview). Dan-Glauser and Gross (2011), Gross and Levenson (1993), and Gross and Levenson (1997) used ANOVAs to detect the effect of emotion regulation on physiological data. Dan-Glauser and Gross (2013) showed in their study, by applying cross-correlations, that synchrony within the physiological channel decreased, if participants were instructed to suppress their emotions. Lacey and Lacey (1958), on the other hand, showed that the physiological response can also vary within an individual (see also Cacioppo et al., 1992). As described in the previous section, basic emotion theory, as well as some of the appraisal approaches, suggest a higher intra-individual synchrony during emotion. Nevertheless, some responses, e.g., respiratory and cardiovascular measures (respiratory sinus arrhythmia; RSA) are also synchronized in order to assist biological functions within our body (Yasuma, 2004; Ben-Tal et al., 2012; Garcia et al., 2013).

In contrast to the large number of studies that examine the correlates of emotional response systems to emotional stimuli (Rosenberg and Ekman, 1994; Calvo and Miguel-Tobal, 1998; Reisenzein, 2000; Bonanno and Keltner, 2004; Mauss et al., 2005; Sze et al., 2010; Hsieh et al., 2011; Dan-Glauser and Gross, 2013), there are only a few empirical studies that provide a quantitative measure of synchrony (Wenger, 1942; Lacey and Lacey, 1958; Lazarus et al., 1963; Nesse et al., 1985; Kettunen et al., 1998). There may be several reasons for this: Firstly, the analysis of physiological response variables is difficult, since they require multivariate, nonlinear, and non-stationary analysis methods (Zong and Chetouani, 2009). Non-stationarity arises when the joint probability density function (pdf) of the response variables changes over time (see next section for more details). Most of the approaches applied so far e.g., cross-correlation, implicitly rely on the stationarity of the physiological signals, and such an assumption is not fulfilled in practice (Muma et al., 2010).

Secondly, specifying a model that quantifies a time-varying synchrony of multiple response variables is not trivial (Dan-Glauser and Gross, 2013).

Thirdly, not only the psychophysiological responses, but also the emotions, impose additional challenges and the demand for sophisticated analytical methods. Emotions have often been treated as static phenomena, similar for different individuals, and have been analyzed by using nomothetic approaches, neglecting the intra-individual variability and the dynamic process of an emotional experience (Kuppens et al., 2009). Inter-individual analysis of the mean values of physiological responses overlooks the possible synchronous response within an individual over time. Therefore, an intra-individual analysis is more appropriate for the analysis of synchrony (Lazarus et al., 1963; Mauss et al., 2005; Hsieh et al., 2011). In the following section, a new approach, which takes the non-stationarity of the data into account, is introduced for analyzing multivariate synchrony of peripheral physiological measures in an individual during an emotional event.

## A new approach for the quantification of synchrony of multivariate psychophysiological signals

In this section, we introduce a new time-frequency based latent variable approach for the quantification of the synchrony of peripheral physiological responses, such as the activity of the heart, respiration, and the electrodermal activity level. The concepts of spectral bivariate coherence and time-frequency bivariate coherence from a signal processing perspective are first discussed. These concepts provide the basis for the quantification of synchrony of non-stationary psychophysiological response variables. Bivariate coherences are used as indicators of a latent state-space model, which quantifies one latent synchronized variable^1^.

### Spectral bivariate coherence

A frequently used function to examine the linear relation at frequency *f* between two signals *x*(*t*) and *y*(*t*), which are a function of time *t*, is the spectral coherence *C*_*XY*_(*f*) (Marple, 1987; Brillinger, 2001), which is defined according to

(1) $$C_{XY}(f)\;=\frac{S_{XY}(f)}{\sqrt{S_{XX}(f)S_{YY}(f)}}.$$

Here, *S_XY_*(*f*), *S_XX_*(*f*), and *S_YY_*(*f*) denote the cross-spectrum and the auto-spectra of *x*(*t*) and *y*(*t*), respectively. The signals *x*(*t*) and *y*(*t*) can, e.g., be two different psychophysiological time series measurements. In practice, spectra can be estimated, e.g., based on the periodogram, which is computationally efficient, since it uses the fast Fourier transform (FFT; Brigham, 2002). The raw periodogram is not a consistent spectral estimate, since its mean-squared error does not decrease to zero as the number of samples used in the computation increases to infinity (Marple, 1987). Consistent spectral estimates can be obtained, e.g., by Welch's and Bartlett's methods, by approaches that: (i) split the original measurement into *K* segments, for which the periodogram is computed and (ii) obtain the overall spectral estimate by averaging over the *K* periodograms of the segments. Alternatively, consistency can be achieved by the Blackman-Tukey estimator which performs a smoothing of the periodogram. This is achieved by a convolution of the periodogram with a spectral window to reduce the variance. In the time-domain, this operation corresponds to a multiplication of the sample covariance sequence with a lag window of smaller size than the data size (Stoica and Moses, 2005).

*C_XY_*(*f*) displays the consistency-of-phase-relationship between *x*(*t*) and *y*(*t*) and provides a frequency selective measure of the phase coupling between the two signals (Brillinger, 2001).

**Figure 1:**
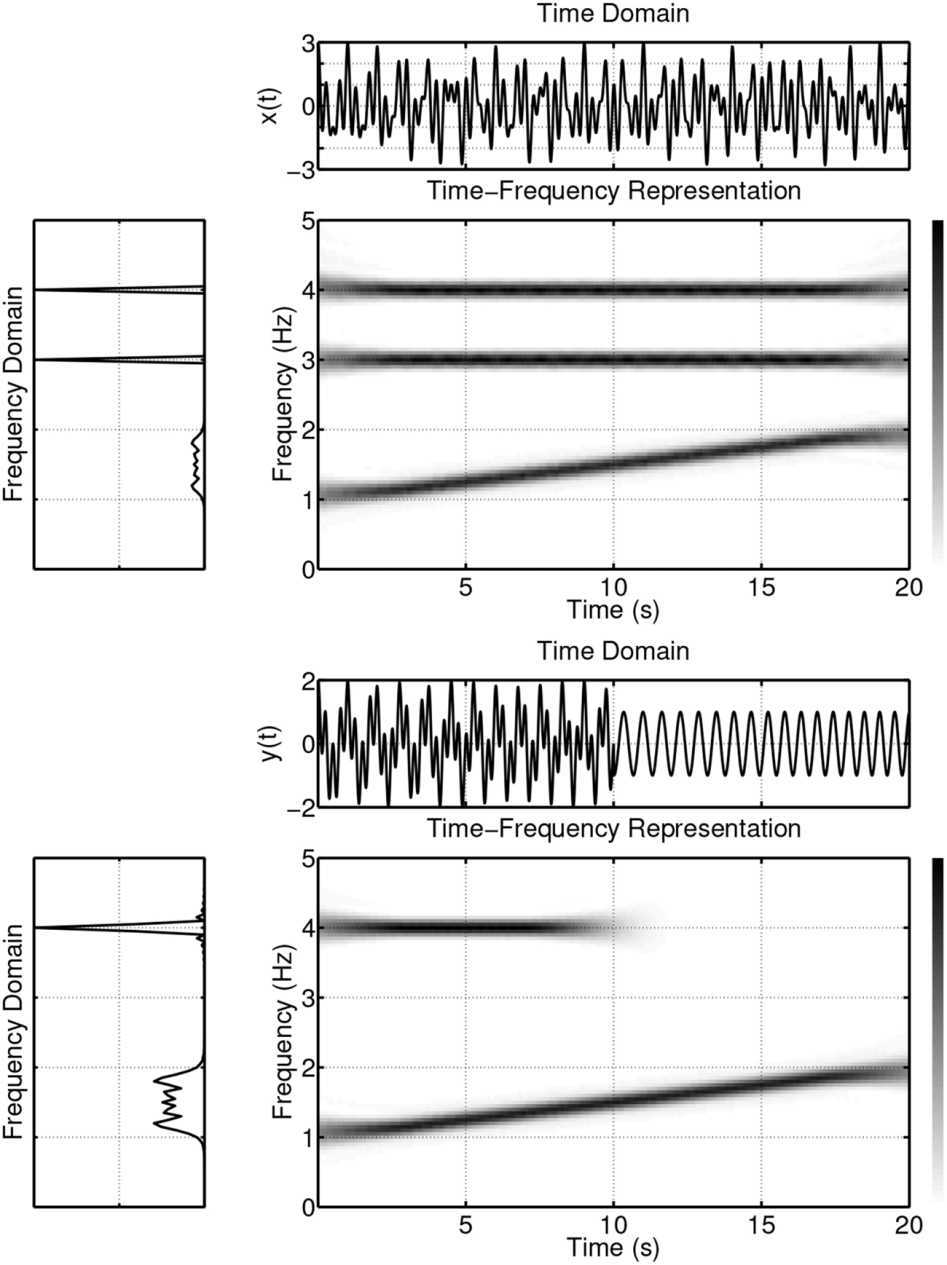
**Example of two non-stationary signals in time, frequency and time-frequency domains**.

The values of the magnitude squared coherence |*C_XY_*(*f*)|^2^ will always satisfy the relationship 0 ≤ |*C_XY_*(*f*)|^2^ ≤ 1. Since *C_XY_*(*f*) is normalized by the product of the auto-spectra (see Equation 1), it is independent, e.g., of different amplitudes of *x*(*t*) and *y*(*t*). If *x*(*t*) and *y*(*t*) are completely uncorrelated, their coherence will be zero. If, on the other hand, |*C_XY_*(*f*)|^2^ = 1 then *y*(*t*) can be fully predicted from *x*(*t*) by a linear and time-invariant system (Marple, 1987). The case |*C_XY_*(*f*)|^2^ < 1, as described in Bendat and Piersol (1990), may be either due to: (i) noise entering the measurements, (ii) a non-linear functional relationship between the signals, or (iii) further inputs in addition to *x*(*t*) contributing to the output. It should be emphasized, however, that the above mentioned statements about *C_XY_*(*f*) are only valid for second order stationary signals (Marple, 1987; Bendat and Piersol, 1990; Brillinger, 2001). Second order stationarity implies a constant mean and variance, as well as an autocovariance function that does not depend on *t*. To overcome the limitations that arise from the assumption of stationary signals, coherence measures for non-stationary signals have been developed. These are based on the wavelet transform (Grinsted et al., 2004) and time-frequency distribution based methods (White and Boashash, 1990; Matz and Hlawatch, 2000; Muma et al., 2010; Orini et al., 2011). The following example illustrates some limitations of classical spectral coherence analysis and provides motivation for the use of time-frequency representations. Figure 1 displays the time, frequency, and joint time-frequency representations of two non-stationary signals *x*(*t*) and *y*(*t*).

- *x*(*t*) consists of a superposition of a linear frequency modulated signal (a signal that consists of a single frequency component whose instantaneous frequency changes linearly over time; starting at 1 Hz for *t* = 0 and reaching 2 Hz at *t* = 20 s) and two sinusoidal signals with frequencies of 3 Hz and 4 Hz.
- *y*(*t*) consists of a superposition of a linear frequency modulated signal (an instantaneous frequency starting at 1 Hz for *t* = 0 and reaching 2 Hz at *t* = 20 s) and a sinusoidal signal with a frequency of 4 Hz and of 10 s duration.

**Figure 2:**
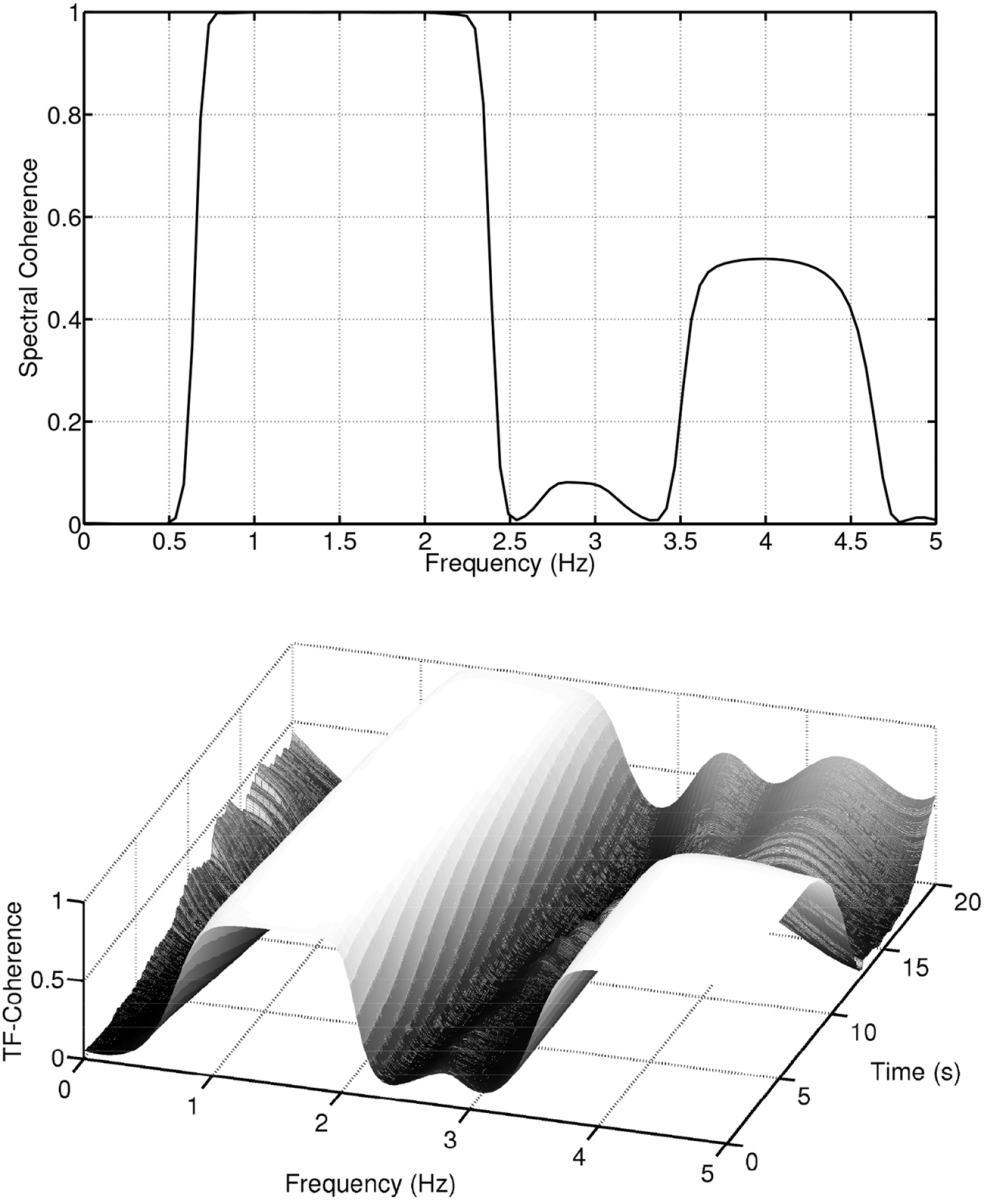
**Spectral coherence and time-frequency coherence of the simulated non-stationary signals**.

The time-frequency representation, like a sheet of music, displays the frequency content of a signal evolving over time. This wealth of information is lost when only the frequency domain is considered, since in the spectrum estimation process, averaging over time is performed. The loss of information is passed on to the spectral coherence measure *C_XY_*(*f*). Figure 2 (first panel) displays the spectral coherence estimate for the above example. *C_XY_*(*f*) only indicates that there is a high synchrony between *x*(*t*) and *y*(*t*) in the frequency region between 1 and 2 Hz, but the evolution/progression over time is averaged out. Furthermore, *C_XY_*(*f*) takes a value of about 0.5 at a frequency of 4 Hz, suggesting a moderate coupling between the signals. This average value neglects the fact that the coherence is nearly equal to one for half of the time and zero for half of the time. These examples illustrate, if the signals are non-stationary, i.e., their spectra evolve over time, that it is important to take the time variation information into account when establishing measures of coherence.

### Time-frequency bivariate coherence

The notion of time-frequency (TF) coherence was first defined by White and Boashash (1990) and then extended by Matz and Hlawatch (2000). The definition is

(2) $$C_{XY}(t,f)\;=\frac{S_{XY}(t,\;f)}{\sqrt{S_{XX}(t,\;f)S_{YY}(t,\;f)}},$$

where *S_XY_*(*t, f*), *S_XX_*(*t, f*) and *S_YY_*(*t, f*) are the cross and auto time-frequency distributions of *x*(*t*) and *y*(*t*), respectively. There exist some conditions, which are stated in Matz and Hlawatch (2000), that are necessary for *C_XY_*(*t, f*) to be well defined and produces meaningful results. In particular, these conditions guarantee that 0 ≤ |*C_XY_*(*t, f*)|^2^ ≤ 1, where |*C_XY_*(*t, f*)|^2^ = 0 for uncorrelated signals and |*C_XY_*(*t, f*)|^2^ = 1 if *x*(*t*) and *y*(*t*) are related via a linear time-invariant filter of sufficiently short length compared to the stationarity width (for details see White and Boashash, 1990).

**Figure 3:**
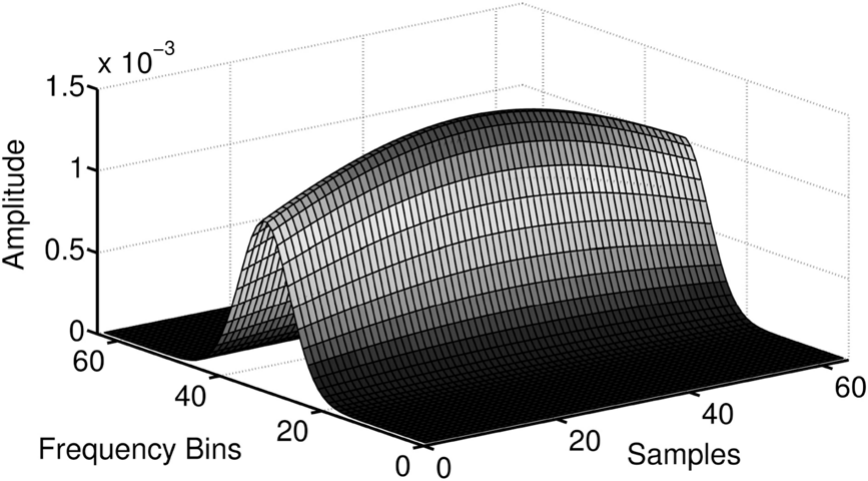
**The implementation of the Gaussian smoothing kernel**. Smoothing of the time-frequency distributions is necessary for *C_XY_*(*t, f*) to be well defined and produce meaningful results.

The time-frequency distribution used in this paper, is the spectrogram which satisfies the conditions for *C_XY_*(*t, f*) to be well defined and has been suggested for use in TF coherence estimation by White and Boashash (1990). The spectrogram is based on the short-term Fourier transform (STFT), and is simply a sequence of FFTs of windowed data segments, where the windows overlap in time. The spectrogram is defined as the magnitude square of the elements of the STFT. The spectrogram yields a time-frequency plot which contains columns of spectral estimates for a specific moment in time. In addition to the choice of the nature of the time-frequency distributions that underpin *S_XX_*(*t, f*), *S_YY_*(*t, f*) and *S_XY_*(*t, f*), it is also necessary to perform a smoothing operation on the distributions, (Matz and Hlawatch, 2000). In this paper, as in Muma et al. (2010), the smoothing is performed by a 2 dimensional filtering with a Gaussian kernel as shown in Figure 3. The parameters of the Gaussian kernel define the time and frequency resolution capability of *C_XY_*(*t, f*) and have been chosen such that both sharp changes in time (time resolution) and individual frequency regions of interest (frequency resolution) can be resolved. Figure 2 (second panel) illustrates the resolution capabilities based on the signals displayed in Figure 1. It can be seen that *C_XY_*(*t, f*) is able to display the time-varying coherence of the two non-stationary signals. As discussed earlier, this is not possible with the spectral coherence *C_XY_*(*f*), alone. Similarly, time domain methods, that rely on stationarity, e.g., correlation coefficients, cannot adequately describe the time-varying relationship between these two exemplary non-stationary signals. In particular, computing the correlation coefficient relies on a non time-varying correlation function (i.e., stationarity).

**Figure 4:**
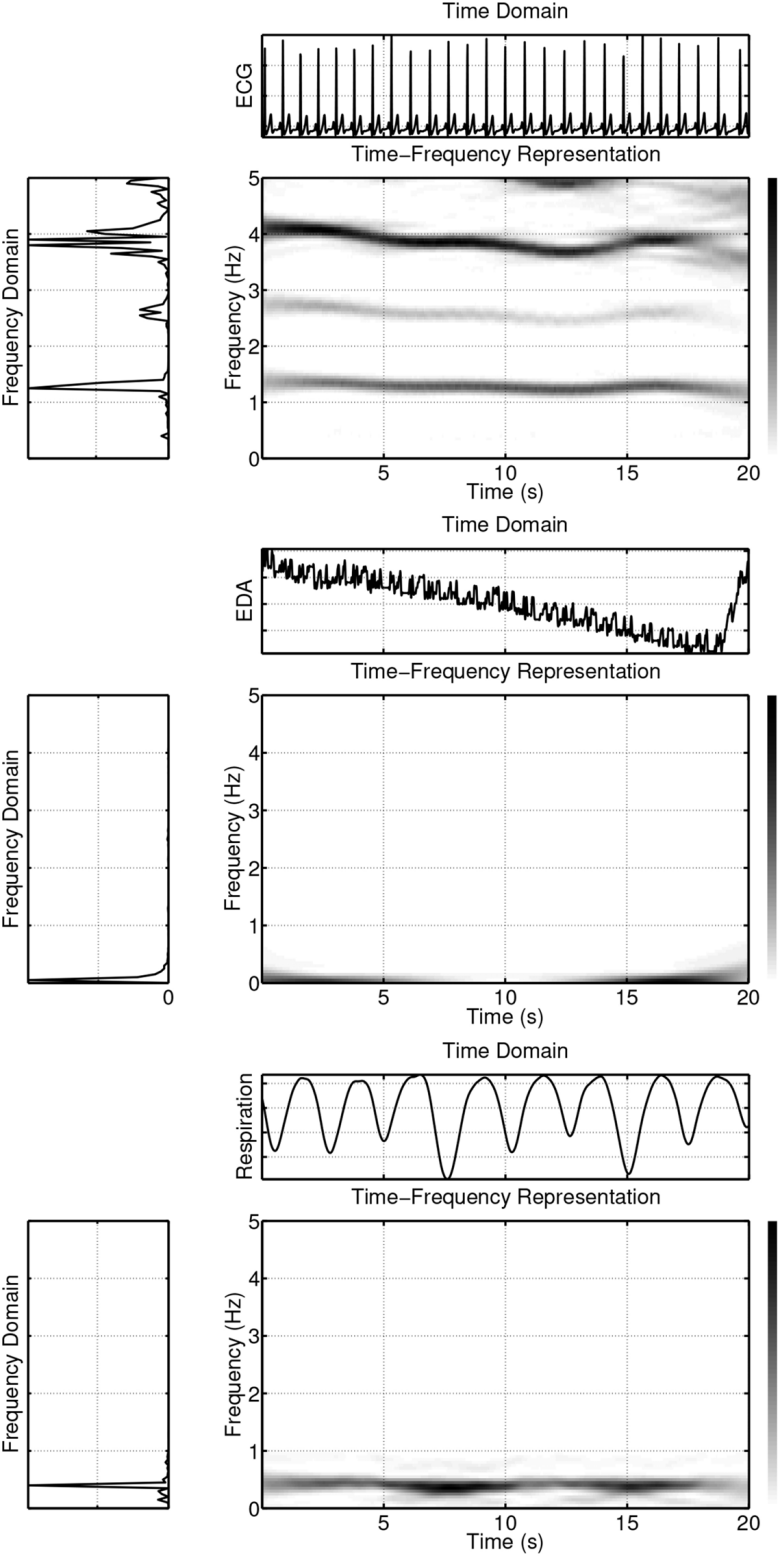
**Example of ECG, EDA and respiration signals in time, frequency and time-frequency domains**.

To illustrate the usefulness of time-frequency based methods for analyzing psychophysiological data, three synchronously measured signals have been considered: An electrocardiogram (ECG) signal, a respiratory signal, and a signal measuring the electrodermal activity level (EDA). The results are shown in Figure 4. Visual inspection quickly reveals that the spectra change over time, indicating the non-stationarity of the signals. A more formal testing for stationarity of some physiological signals, among them ECG signals and respiratory signals, has been performed in Muma et al. (2010) using a frequency domain test, suggested by Brcich and Iskander (2006). The test is able to determine if stationarity exists at a given frequency region of interest. The test, in general, rejected the hypothesis of stationarity for ECG and respiratory signals.

**Figure 5:**
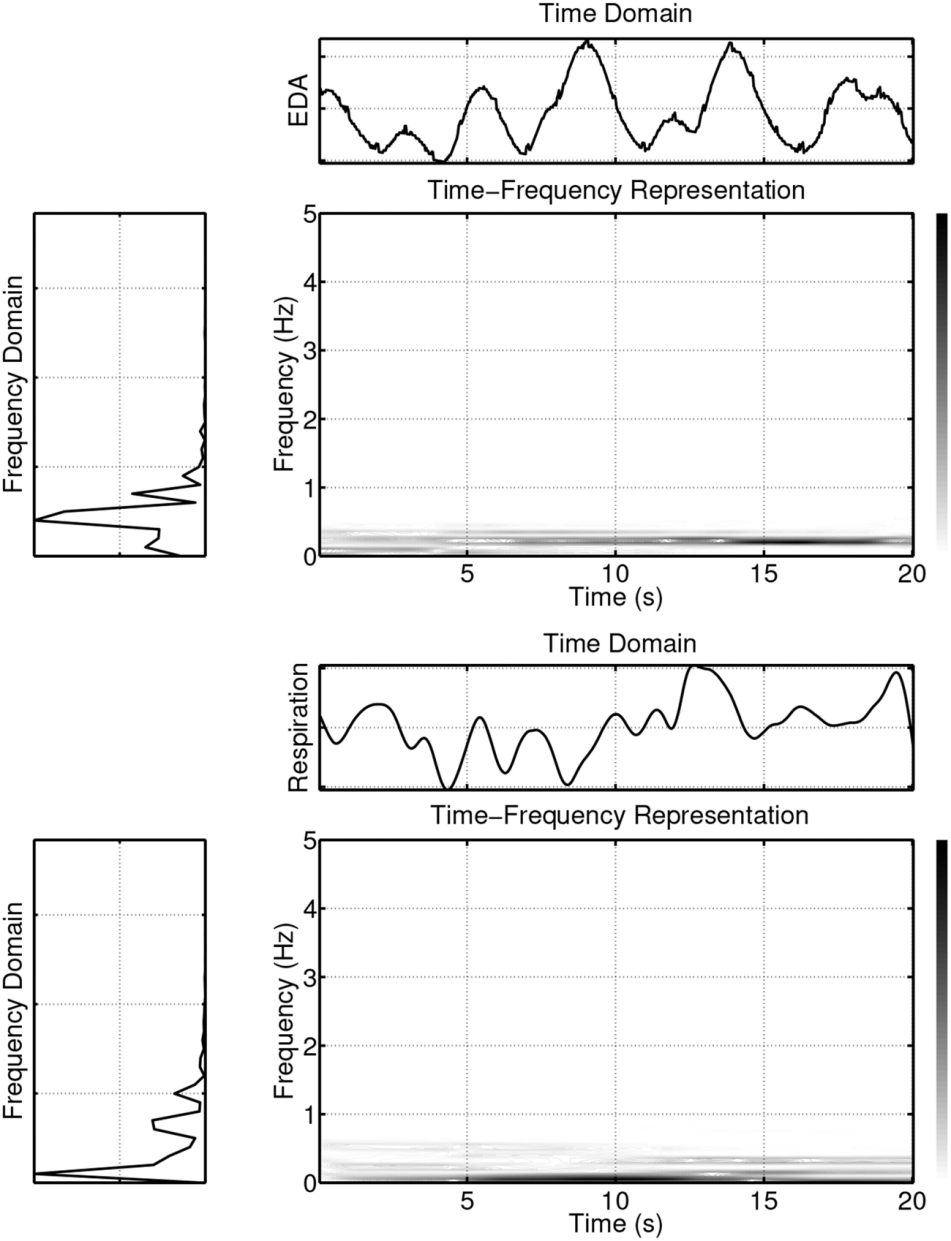
**Example of synchronously measured EDA and respiration signals that both contain a cyclic component at the frequency region of approx. 0.4 Hz**.

**Figure 6:**
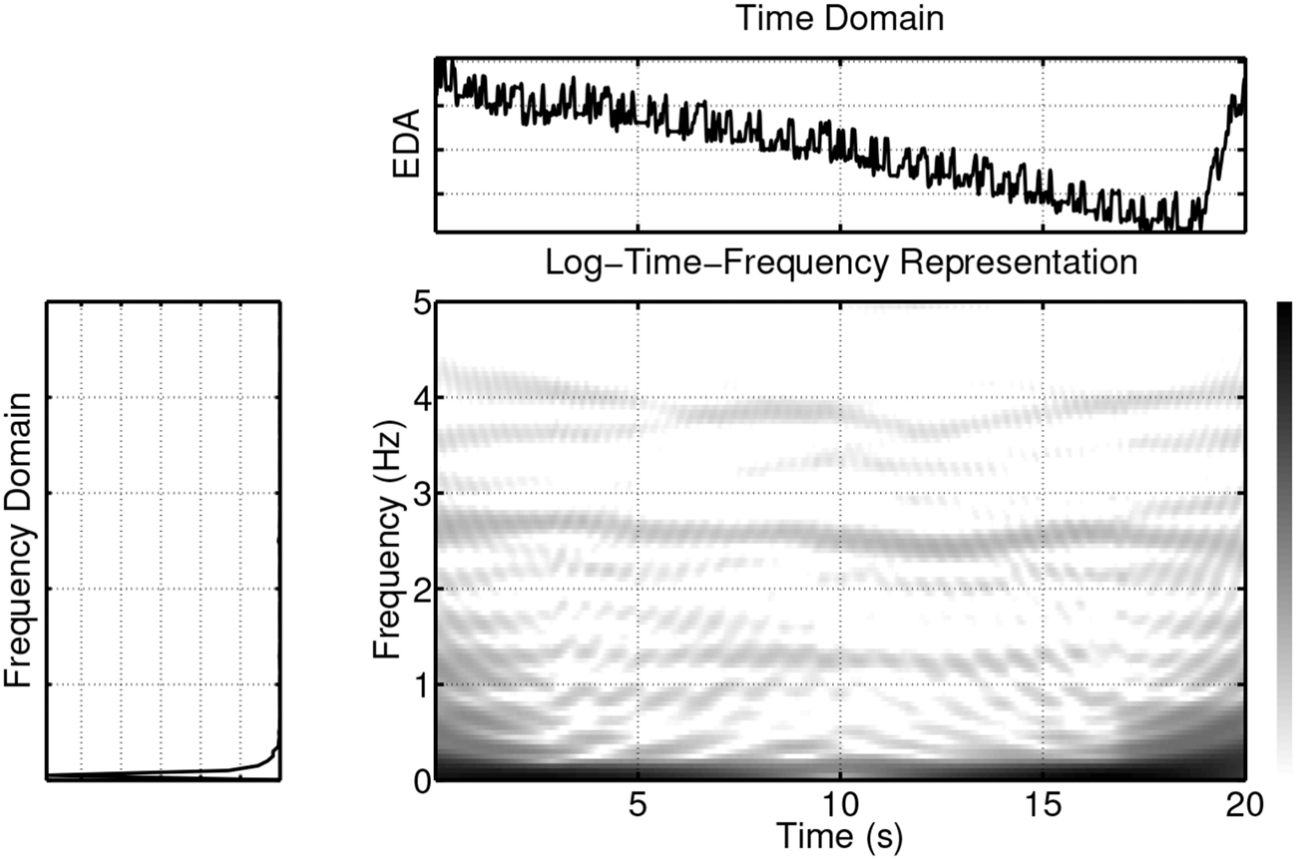
**Element-wise logarithm of the time-frequency distribution of the EDA signal depicted in Figure 4, second panel**. For this example, the pulse frequency region and its harmonics become clearly visible in the EDA signal.

As can be seen in Figure 4, the power of the signals is not evenly spread out in the time-frequency plane, but instead is concentrated in delineated regions. If only one such region exists, the signal is referred to as “mono-component,” while if multiple delineated regions exist, the signal is referred to as “multi-component.” For example, a linear frequency modulated signal is a mono-component signal. An ECG signal is a multi-component signal, which consists of the pulse frequency region (varying between about 1.2 and 1.4 Hz for the example shown in Figure 4, first panel) and its harmonics, which are located in regions given by integer multiples of the pulse region. The power of the EDA (Figure 4, second panel) and respiration (Figure 4, third panel) signals is primarily concentrated in one region, with only a small amount of power in other regions. Unlike the ECG and respiration signals, the EDA, in general, does not have a cyclic nature, but contains trends and abrupt changes. Most of its signal power is concentrated in the lowest frequencies (see Figure 4, second panel) and forms the delineated region of the EDA in the time-frequency plane. However, analysis of the EDA data showed that the EDA signal also contains power in other regions during some time intervals. For example, frequency domain analysis shows that both the EDA data of Figure 5 (first panel) and the synchronously measured respiration signal Figure 5 (second panel) contain power in the region around 0.4 Hz. Coherence analysis can reveal whether this is a coincidence. An interesting question is if or when this coherence occurs and whether the method of coupling depends on an emotional state. The existence of a small amount of power in higher frequency regions of the EDA signal, is evident in Figure 6 where the element-wise logarithm of the time-frequency distribution of the EDA signal depicted in Figure 4, second panel, is shown. For this example, the pulse frequency region and its harmonics become clearly visible in the EDA signal. For a thorough analysis of EDA signals, the interested reader is referred to Lim et al. (1997).

**Figure 7:**
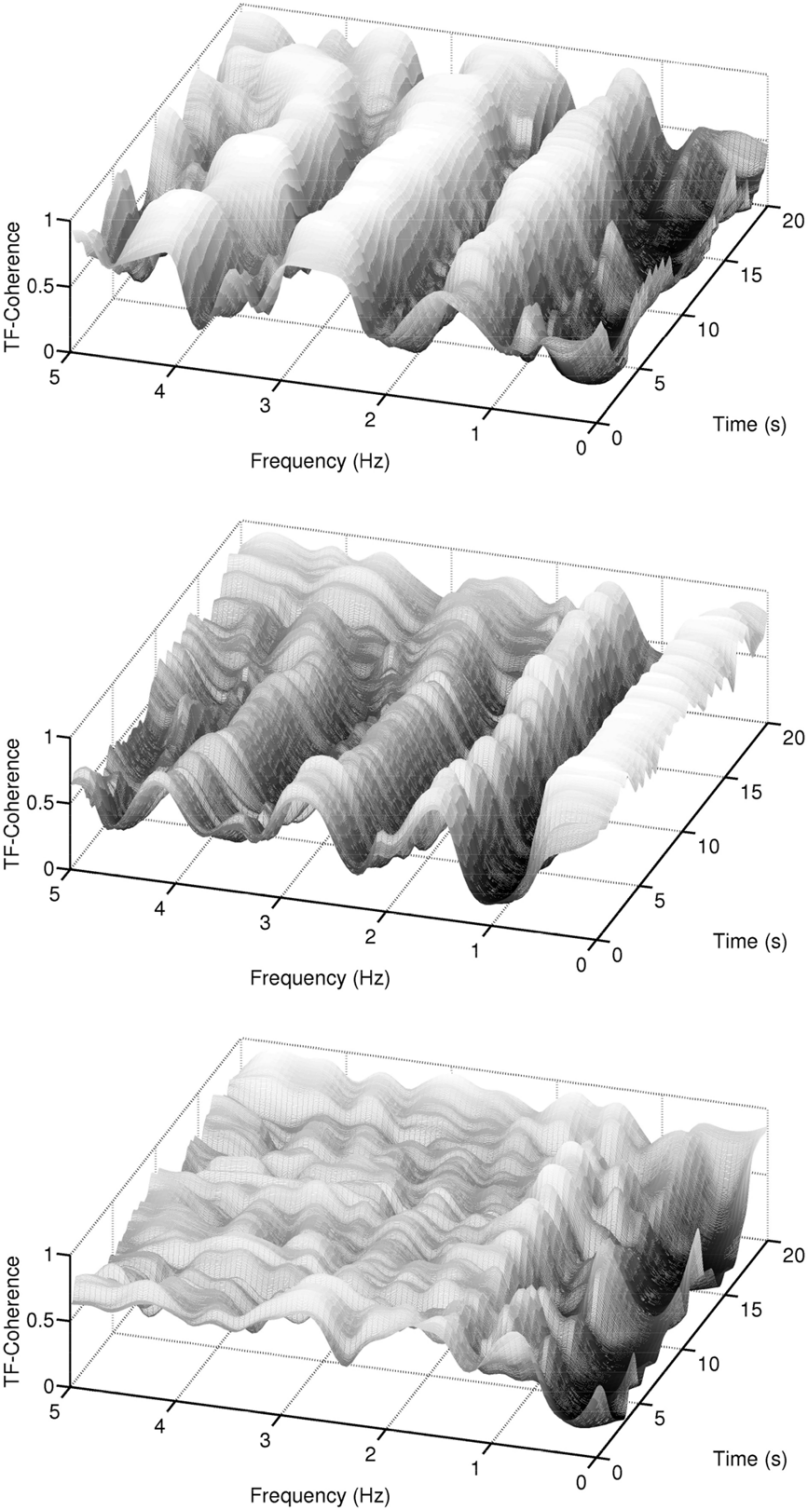
**Example of coherences between ECG, EDA, and respiration signals in the time-frequency domain**. The first panel shows the coherence between the ECG and EDA signals, the second panel shows the coherence between the ECG and respiration signals, and the third panel shows the coherence between the EDA and respiration signals.

**Figure 8:**
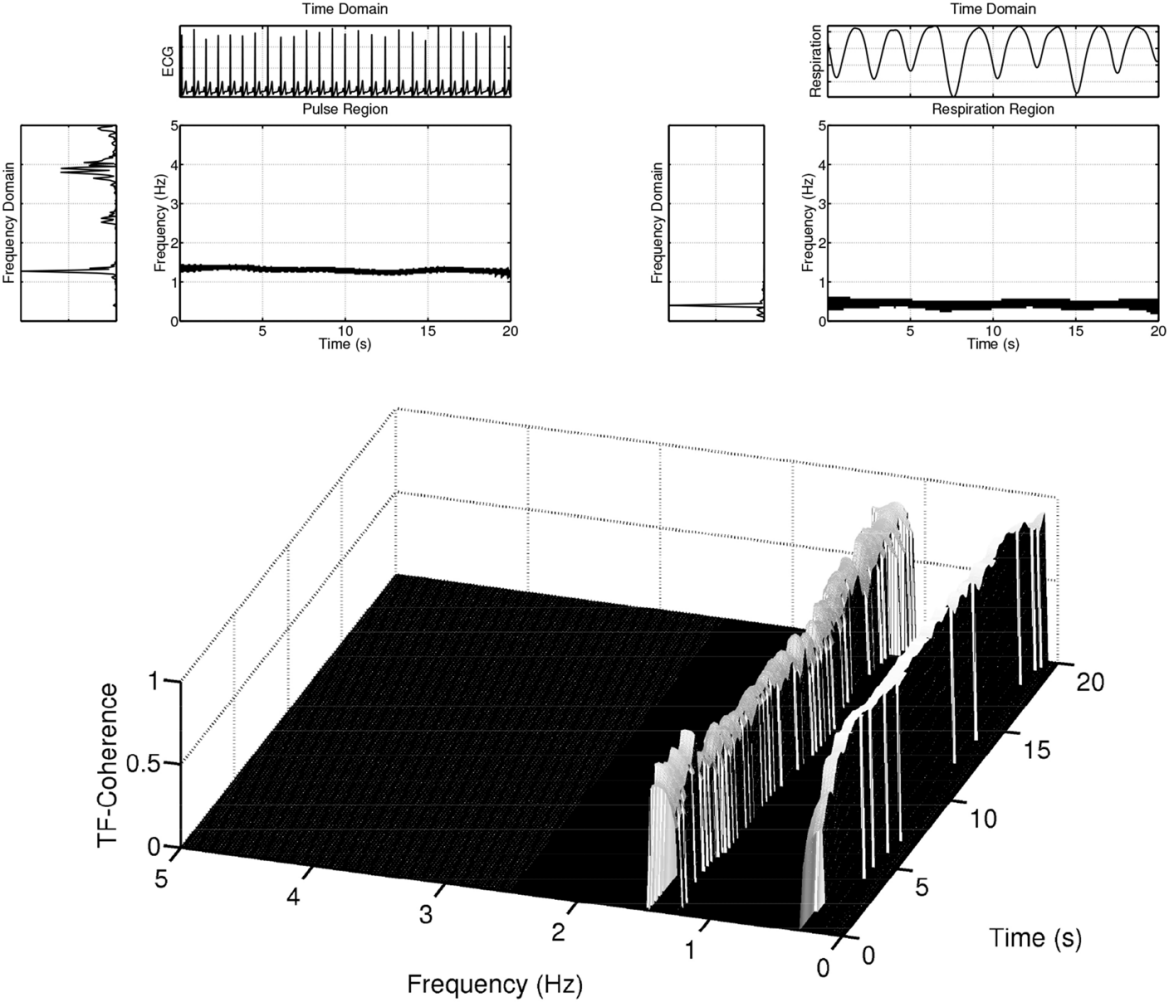
**Example of coherences within delineated regions, i.e., the pulse and respiration regions, between ECG and respiration signals**.

**Figure 9:**
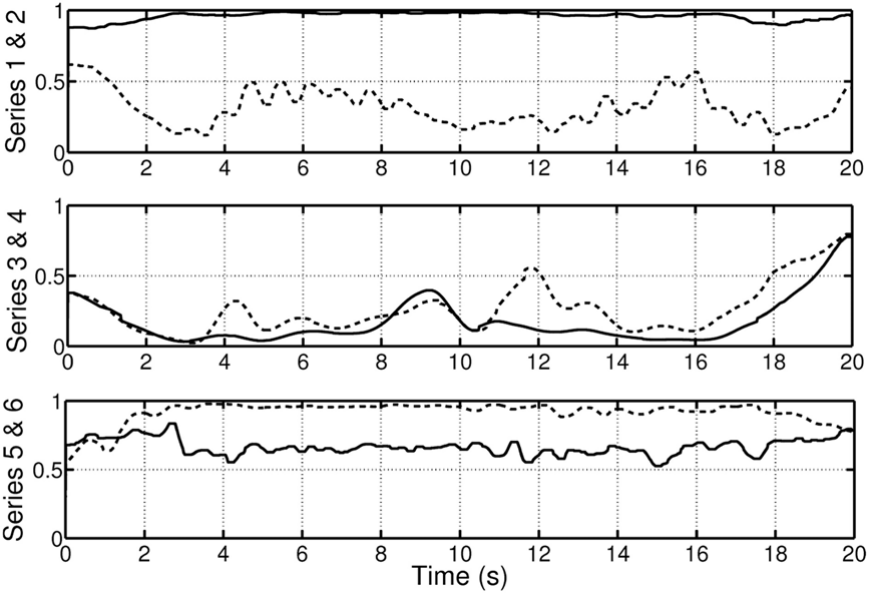
**Example of the pairwise coherence measure based on the concept of delineated regions in the time-frequency plane as shown in Figure 8**. Series 1 & 2 plots the coherence of the ECG and EDA signals in the pulse region (solid) and EDA region (dashed), respectively. Series 3 & 4 depict the coherence of the respiration and EDA signals in the respiration region (solid) and EDA region (dashed). Series 5 & 6 depict the coherence of the respiration and ECG signals in the pulse region (solid) and respiration region (dashed).

Figure 7 plots the pairwise coherences (as given by Equation 2) of the three signals. For this example, the coherence between the ECG and EDA signals (first panel) is “high” in the pulse frequency region and its harmonics, while it is “low” in the frequency region that contains most of the EDA signal power. The coherence between the ECG and respiration signals (second panel), on the other hand, takes maximal values at the respiration frequency region, is “moderate” at the pulse frequency region and its harmonics, and “low” elsewhere. The coherence between the EDA and the respiration signals (third panel) is low for the respiration and EDA frequency regions and “moderate” elsewhere. Instead of a narrative description of the pairwise relationships, we briefly describe a scalar measure which quantifies this information. For this, the concept of coherences within delineated regions in the time-frequency plane, as detailed in Muma et al. (2010), is followed. Figure 8 shows the pulse and respiration frequency regions. Muma et al. (2010) proposed an algorithm to detect these regions within the time-frequency plane. The idea of the algorithm, in brief, is: First, the frequency of the auto-spectrum that contains maximal energy *f*_max_, 0 is determined for a data segment, in our case, of length 10 s. The frequency of maximal energy *f*_max_(*t*) of the non-stationary signal varies around *f*_max_, 0 and can be found by searching near the maximal value of the periodogram at each time instant. The delineated regions include all neighboring frequencies for which the power drops less than 3 decibel compared to *f*_max_(*t*). By applying a mask onto *C_XY_*(*t, f*), which is equal to one within the detected regions and zero elsewhere (see Figure 8), analysis is restricted to the regions of interest. Note that, unlike classical spectrum based heart rate variability (HRV) analysis, (e.g., Niskanen et al., 2004; Acharya et al., 2006) and references therein, fixed frequency bands (VLF, LF, HF), which are set a-priori and remain constant over time, are not assumed. Our algorithm automatically adapts the frequency region of interest over time to the data at hand. Based on this, scalar time-varying coherence measures between two signals, such as, for example, the average coherence at a frequency region of interest for each time instant, can be determined. Figure 9 plots the pairwise (bivariate) coherence measure for the three signals in the respective frequency regions. It is clearly visible from these examples that a coupling between the signals exists and that the amount of synchronization varies over time. In the next section, a system-wise coherence measure based on the pairwise coherences, is defined.

### A latent state space modeling approach to a system-wise synchrony measure

In this subsection, the well-known state space modeling approach (e.g., Durbin and Koopman, 2001) is used to specify an overall synchrony measure. The basic idea is to use the multivariate time series of pair-wise coherence measures within delineated frequency regions (see previous subsection and Figure 9) and to specify a measurement model that operationalizes a latent variable which represents an underlying state of an overall system-wise synchrony. In addition to the measurement model, a structural model describes the regression of a state relative to its previous states.

For a given individual *i*, the specification of the measurement and structural model is given in the so-called non-innovation form (e.g., Gilbert, 1993):

(3) $$z_{it}=H_{t}\xi_{it}+R_{t}e_{it}$$

(4) $$\xi_{it}=F_{t}\xi_{i(t-1)}+G_{t}u_{it}+Q_{t}\eta_{it}$$

In the measurement model (Equation 3), we assume that a given *p*-dimensional observed variable *z_it_*, at time point *t*, can be regressed on a *q*-dimensional (latent) state vector ξ_*it*_, where *e_it_* is a *p*-dimensional residual vector (white noise). The variable *z_it_* hereby includes the pair-wise coherence measures in selected frequency regions (delineated regions), whereas ξ_*it*_ represents the overall synchrony for an individual *i* at time point *t*, which is the variable of interest. The estimation of the pair-wise coherence measures is described in the previous section. *H_t_* and *R_t_* are time-varying coefficient matrices, respectively of dimensions (*p* × *q*) and (*p* × *p*) that reflect the time-varying relationship between the latent variable vector ξ_*it*_ and the observed variable vector *z_it_*, and the time-varying relationship between the residual vector *e_it_* and the observed variable vector *z_it_*, respectively. In the structural model (Equation 4), it is assumed that the state vector ξ_*it*_ can be regressed on a previous state vector ξ_*i*(*t* − 1)_ and on a *m*-dimensional covariate vector *u_it_* (e.g., an intervention), where η_*it*_ is a *q*-dimensional latent residual (white noise) vector. Again, *F_t_* (*q* × *q*), *G_t_* (*q* × *m*), and *Q_t_* (*q* × *q*) are time-varying coefficient matrices. The coefficient matrix *H_t_* indicates the time-dependent strength of the relationship of the state variable ξ_*it*_ and its indicators/measures.

Predictions of latent states (ξ_*i*(*t* + 1)_) can be conducted, for example, by applying the Kalman filter (e.g., Grewal and Andrews, 2001; Shumway and Stoffer, 2006). By using a smoothing algorithm (e.g., Durbin and Koopman, 2001), scores for the latent variable ξ_*it*_ can be derived for the time series. The model parameters can be, for example, determined via a maximum likelihood estimator and imposing additional distributional assumptions (for example, normality of the latent variable ξ_*it*_). It is not possible to freely estimate all the parameters in Equations (3) and (4). In order to have an identified model, the typical additional restrictions of latent variable modeling (e.g., scaling variables) are needed (Bollen, 1989). For a given individual *i* the time-dependent pattern of the parameters reflects the changing structural relationship of the individual reactions in the observed variables over the course of time; specifically, here, the psychophysiological responses (e.g., Marwitz and Stemmler, 1998). The time series of the resulting latent states ξ_*it*_ represents the course of the overall system-wise synchrony.

**Figure 10:**
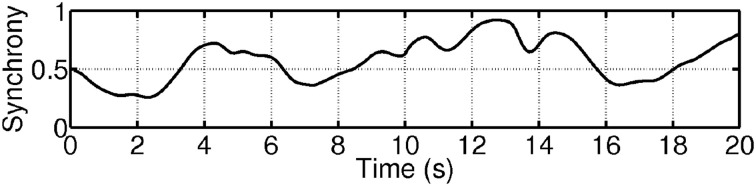
**Example of an overall synchrony measure while a participant was watching a neutral picture and a disgust eliciting picture for 10 s each**.

Figure 10 gives an example of a time series of the system-wise synchrony measure (latent state variable ξ_*it*_) during an emotional episode. The first 10 s (100 points) show the synchrony while a participant was watching a affective neutral picture (barstool: #7025) of the International Affective Picture System (IAPS; Lang et al., 2008). During the last 10 s (100 points) the participant was confronted with a disgust eliciting picture (half ripped-off finger: #3150). As a descriptive result, it can be seen the overall synchrony measure was, on average, higher during the disgust eliciting picture than during the neutral picture.

## Application of two approaches for the quantification of synchrony using data from an emotion regulation study

In this section, we illustrate the application of the proposed measure of synchrony, and the application of a measure proposed by Hsieh et al. (2011), to data from a larger emotion regulation study where psychophysiological signals were collected while participants were watching funny film clips. First, a short description of the larger study, is given. Second, assuming that the induction of emotions leads to person-specific changes in synchrony of the psychophysiological signals, the time series of the overall synchrony measure is analyzed by applying the proposed approach. Third, the results are compared with those obtained by using the approach for the quantification of the (aggregated) synchrony of Hsieh et al. (2011).

### Description of the emotion regulation study

#### Participants

The sample of the emotion regulation study consisted of 58 German male undergraduate students of engineering. The mean age of the overall sample was 23.11 years (*SD* = 3.72). The procedure was fully explained to participants before written informed consent was obtained. Participants took part in a lottery where they could win a portable media player.

#### Procedure

Participants watched a random sequence of two funny clips (each 10-min long), which were cuts of the German version of the slapstick comedy film “You Don't Mess with the Zohan,” starring Adam Sandler. In a small pilot-study, ratings of the clips showed that one film clip was slightly funnier than the other. Blood pressure was measured 5 and 10 min after each clip. Physiological measures were obtained continuously, and self-reported ratings of experienced amusement were collected for each film clip.

#### Measures

The data consisted of physiological and subjective responses during the film clips. The subjective responses are not considered here. Physiological measures were continuously sampled with a frequency of 256 Hz. The following signals were obtained using a BIOPAC MP150 System (Biopac Systems Inc., 2011): ECG, EDA, and respiration signals. In addition to these continuous signals, discrete measures of systolic and diastolic blood pressure were taken every 5 min. In this article, analysis was restricted to the ECG, the EDA, and the respiration signalw. Motion artifacts in the ECG signals were removed using a method proposed by Strasser et al. (2012).

### Application of the proposed approach for the quantification of synchrony

**Figure 11:**
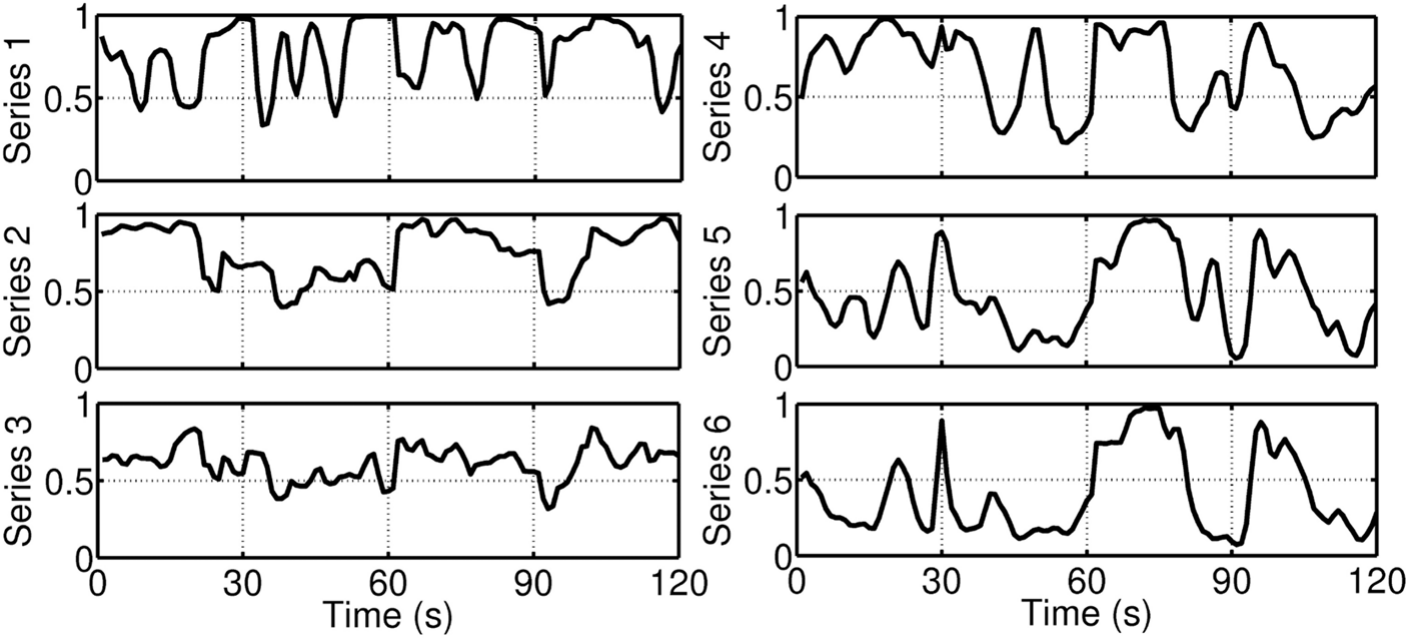
**Example of six time-frequency based pairwise coherence measures (series) for one individual watching funny film clips**. Series 1: ECG-EDA coherence (EDA region), Series 2: ECG-EDA coherence (ECG region), Series 3: RE-ECG coherence (ECG region), Series 4: RE-ECG coherence (RE region), Series 5: RE-EDA coherence (EDA region), Series 6: RE-EDA coherence (RE region). During the first 60 s, the participant was watching a film clip, which was rated as moderately funny. During the last 60 s, the participant was shown a funnier film clip.

In order to obtain an overall quantity of the synchrony, the following two step procedure was applied. First, the time-frequency based pairwise coherence measures were obtained. Using three signals (here: ECG, EDA, and respiration) results in six pairwise measures (two measures for each pair of physiological signals; see for example Figure 9). Figure 11 gives an example of the six resulting time series for one participant over a period of 120 s. During the first 60 s, the participant was watching the film clip, which was rated as moderately funny. During the last 60 s, the participant was shown the funnier film clip. As can be seen from Figure 11, some of the pairwise measures are very similar (for example Series 5 and 6).

**Figure 12:**
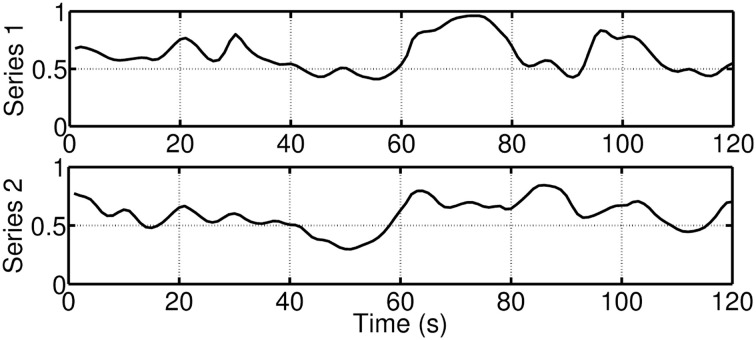
**Example for two overall synchrony measures for two participants**. During the first 60 s, the participants were watching a film clip, which was rated as moderately funny. During the last 60 s, the participants were shown a funnier film clip.

Second, the obtained pairwise measures *z*_*it*_ = (*z*_1*it*_, *z*_2*it*_, …, *z*_6*it*_)^T^ were indicators of a single overall latent variable ξ_*it*_ time series of the system-wise synchrony using the state-space modeling approach as described above (cp. Equation 3). For a given person *i*, the measurement variable vector is given as:

(5) $$\begin{array}{l} z_{it}={\left(z_{1it},z_{2it},\ldots ,z_{6it}\right)}^{\text{T}}={\left(a_{1i},a_{2i},\ldots ,a_{6i}\right)}^{\text{T}}\\ \;+\left(h_{1i},h_{2i},\ldots ,h_{6i}\right){\;}^{\text{T}}\xi_{it}+{\left(e_{1it},e_{2it},\ldots ,e_{6it}\right)}^{\text{T}} \end{array}$$

where *a*_1*i*_, …, *a*_6*i*_ represent additional intercepts, *h*_1*i*_, *h*_2*i*_, …, *h*_6*i*_ are loadings, and *e*_1*it*_, …, *e*_6*it*_ ~ *N*(0, σ_*e*_). *N*(0, σ_*e*_) is the zero Gaussian distribution with a standard deviation equal to σ_*e*_. The latent variable ξ_*it*_ was assumed to be uni-dimensional (see Equation 4), reflecting the overall synchrony. For a given person *i*, the structural model is given as:

(6) $$\xi_{it}=g_{1i}+f_{1i}\xi_{i(t-1)}+\eta_{it}$$

where *g*_1*i*_ is an intercept, *f*_1*i*_ is a regression coefficient, and η_*it*_ ~ *N*(0, σ_η_). Figure 12 provides examples of overall synchrony measures for two participants. Again, during the first 60 s, participants were watching a film clip, which was rated as moderately funny. During the last 60 s the participants were shown a funnier film clip.

Table 1 presents parameter estimates, standard error estimates, and confidence intervals for two participants (see Equations 5, 6). The results between the two participants vary substantially. As can be seen from Table 1, for participant #1 the loadings *h*_1_ and *h*_3_ are not significant and thus the measures *z*_1_ and *z*_3_ are not reliable indicators of synchrony. The other indicators (*z*_2_, *z*_4_, *z*_5_, *z*_6_) indicate the degree of overall synchrony. In contrast, for participant #2 a different set of indicators (*z*_1_, *z*_4_, *z*_5_, *z*_6_) imply an overall level of synchrony. Furthermore, the sign of the loading of indicator *z*_4_ is different, which means that for participant #1 an increase in the overall synchrony leads to an increase in the relationship of the two variables associated with respiration and EDA (*h*_4_ = 0.97), while for participant #2 an increase in the overall synchrony leads to an decrease in the relationship of the two variables associated with respiration and EDA (*h*_4_ = −0.39). These simple examples show that the patterns of synchrony of the psychophysiological signals are very person-specific.

**Table 1:** **Parameter estimates (Est.), standard errors (SE) and 95%-confidence intervals (CI) of the parameter estimates obtained from the estimated latent state space model approach for the quantification of an overall measure of synchrony**.

	**Participant #1**				**Participant #2**			
	**Est**.	**SE**	**low.CI**	**up.CI**	**Est**.	**SE**	**low.CI**	**up.CI**
*h*_1_	0.01662	0.11579	−0.22833	0.23201	1.12273	0.12896	0.84300	1.37206
*h*_2_	0.31343	0.11463	0.07954	0.53663	−0.28423	0.14358	−0.54200	0.01135
*h*_3_	0.23581	0.11447	−0.01626	0.46141	−0.00653	0.15265	−0.29670	0.28018
*h*_4_	0.97394	0.09882	0.77685	1.16253	−0.38696	0.14700	−0.65100	−0.05721
*h*_5_	1.47662	0.09246	1.26250	1.63411	1.37362	0.12055	1.11320	1.58214
*h*_6_	1.53865	0.08974	1.33940	1.69108	1.47724	0.11850	1.21750	1.69180
*a*_1_	0.77227	0.07314	0.63287	0.93054	−0.02784	0.08462	−0.16620	0.16572
*a*_2_	0.56165	0.07300	0.41896	0.71099	0.79885	0.08541	0.61610	0.94672
*a*_3_	0.46500	0.07148	0.32892	0.61555	0.56411	0.09111	0.39330	0.74058
*a*_4_	0.04048	0.06548	−0.07622	0.18146	0.94931	0.08880	0.74230	1.10420
*a*_5_	−0.45378	0.06359	−0.55925	−0.29915	−0.31729	0.08010	−0.43890	−0.11750
*a*_6_	−0.55987	0.06034	−0.65546	−0.42617	−0.44512	0.08180	−0.55910	−0.24040
σ_*e*_	0.02259	0.00126	0.01987	0.02481	0.02853	0.00160	0.02510	0.03135
*f*_1_	0.92437	0.05662	0.73895	0.95801	0.91452	0.05785	0.72670	0.95854
*g*_1_	0.04628	0.03439	0.02541	0.15752	0.04996	0.03255	0.02210	0.14888
σ_η_	0.00357	0.00100	0.00189	0.00566	0.00283	0.00097	0.00120	0.00502
ξ_0_	0.68325	0.09688	0.50251	0.87255	0.79121	0.09453	0.61010	0.99287

*During the first 60 s, the participants were watching a film clip, which was rated as moderately funny. During the last 60 s, the participants were shown a funnier film clip. Confidence intervals were obtained using a (parametric) bootstrap procedure provided by the MARSS package in R-project*.

### Application of a complementary approach for the quantification of the synchrony proposed by Hsieh et al. (2011)

To provide a comparison to existing procedures, the recently proposed method by Hsieh et al. (2011) is applied to the dataset. The method estimates an aggregated system-wise synchrony by creating a stochastic network, where high connectivity corresponds to high synchrony of the *J* = 3 signals (here: ECG, EDA, and respiration signals). First, receiver operating characteristics (ROC) were obtained. For a random variable *X*, the ROC displays the probability of correct detection, i.e., the probability of correctly accepting the null hypothesis, as a function of *x*, vs. the probability of false alarm, i.e., the probability of falsely accepting the null hypothesis, as a function of *x*. In our experiment the null hypothesis (*H*_0_) is associated to the time interval with the moderate funny film clip, i.e., the baseline, while the alternative (*H*_1_) is associated with the funnier (emotionally more activating) film clip. The ROC area *A* is defined as the area between the ROC curve and a diagonal line given by an identity function:

$$A=\int_{0}^{1}(P(H_{0}|H_{0},x)-c(x))\frac{dP(H_{0}|H_{1},x)}{dx}\;dc(x).$$

Here, *c*(*x*) is a linear function, that maps the interval of *x* onto the interval [0, 1], which is equivalent with the diagonal line in this context. *P*(*H*_0_|*H*_0_, *x*) and *P*(*H*_0_|*H*_1_, *x*) are the probabilities of correct detection and false alarm given a threshold at *x*, respectively (Hsieh et al., 2011). For the signals acquired while the participants were watching both film clips, in accordance with Hsieh et al. (2011), are divided into $\frac{K}{2}$ = 12 non overlapping segments. The ROC areas were then calculated by defining *P*(*H*_0_|*H*_0_) as the probability of deciding for a particular film clip given that the participants were actually watching this film clip. In this case, we used the cumulative distribution function (CDF) of the single segments, and *P*(*H*_0_|*H*_1_) as the probability of deciding for a particular film clip given that the other film clip was watched. *P*(*H*_0_|*H*_0_) and *P*(*H*_0_|*H*_1_) are used to form the CDFs of the baseline and of the activation phase, respectively.

In the second step, the Spearman rank coefficients of the ROC areas of 24 non-overlapping blocks were calculated. These were formed by 12 blocks of 50 s each from the emotionally neutral phase and 12 blocks of 50 s each from the emotionally eliciting phases. It should be noted that the acquired signal blocks exhibited a non-stationary character. By calculating the Spearman rank coefficients of the ROC area sequences of the non-stationary signal blocks for each individual and signal-pair, a single scalar value is obtained as an averaged measure of coherence. This scalar does not take into account any stochastic changes of the signals, and, hence, it disregards significant information. Applying this procedure to every subject and signal pair, yields a *J* × *J* rank coefficient matrix for each subject and phase. Applying

(7) $$\hat{p}(h|j,j\prime )=\frac{1}{M}\text{max}\left\{\sum_{m\;=\;1}^{M}1_{\left\{{\hat{\rho }}^{\left(m\right)}(j,j^{\prime })\;>\;h\right\}},\text{​}\sum_{m\;=\;1}^{M}1_{\left\{{\hat{\rho }}^{\left(m\right)}(j,j^{\prime })<-h\right\}}\text{​}\right\}$$

from Hsieh et al. (2011) with the critical value *h* = 0.344 taken from the Spearman rank distribution table (see Zar, 1972; for *n* = 24 and α(1) = 0.05), yields a *J* × *J* matrix, called the partition matrix:

$$\hat{p}(h)=\left[\begin{array}{ccc} - & 0.3820 & 0.4270\\ 0.3820 & - & 0.3146\\ 0.4270 & 0.3146 & - \end{array}\right],$$

Here the 1st, 2nd, and 3rd rows and columns belong to the ECG, EDA, and respiratory signals, respectively.

In the third step, a stochastic network was defined, where the nodes correspond to the ECG, EDA, and respiratory signals, and the edges were defined by values from the partition matrix $\hat{p}$(*h*). To introduce randomness, a matrix *u* was formed whose entries were generated by the uniform distribution on the interval [0, 1]. In general, an edge between two nodes *j, j*′ was established, if *b*_con_(*j, j*′) = *p*(*j, j*′) > *u*(*j, j*′), i.e., if the Boolean matrix *b*_con_ had a “1” entry at (*j, j*′). If *p*(*j, j*′) ≤ *u*(*j, j*′), there was no connection between *j* and *j*′ nd the correspondent entry in the *b*_con_ matrix was 0. Each node of the stochastic network is either activated or inactive. To test for system-wise synchrony, in the beginning, a node must be selected for activation. After activation, the node sends a signal, via its edges, to other directly connected nodes, which then activate their neighboring nodes. After sending out the signal, the node that was activated in the first place becomes inactive. This activation, and inactivation, of nodes was iterated until (i) all nodes became activated, in which case system-wise synchrony was achieved, or (ii) a system state was obtained for which system-wise synchrony was impossible to achieve. For *J* nodes, this was done *J* times, each time activating a different node at the beginning. In our case of *J* = 3 nodes, system-wise synchrony could only be achieved by a fully connected network. Based on a Monte Carlo simulation with 10,000 repetitions, the stochastic network achieved system-wise synchrony 511 times. Thus, the resulting stochastic network had a probability of about 5.1 % to achieve system-wise synchrony.

Due to the previous averaging over the subjects, the calculated probability gives us an expected value for achieving system-wise coherence for any of the given subjects. It is important to realize that the result of the simulation is not dependent on an individual subject and that the results depend on the assumption of stationarity.

## Discussion

A new approach for the quantification of synchrony of multivariate non-stationary psychophysiological signals has been proposed. After calculating bivariate time-frequency based coherence measures, a state space modeling procedure was applied to obtain an overall measure of synchrony. The approach gives information on the intra-individual level about the course of synchrony of psychophysiological reactions.

### Methodological and substantive considerations

The use of multivariate time series of physiological reactions for the quantification of an overall synchrony has several methodological and substantive implications. Firstly, the approach provides time-sensitive information about the intra-individual level of synchrony. Thus, it is complementary to alternative approaches, which give information on cross-subjects based (aggregated) synchrony. See, for example the approach proposed by Hsieh et al. (2011)^2^.

Secondly, the proposed approach also provides information about the inter-individual difference of the structure of the synchrony measure. Specifically, the person-specific reaction patterns of how the physiological signals are related with the (latent variable) synchrony have been quantified. The factor loadings within the state space approach provide person-specific information on how the simultaneous activation of two given signals, e.g., ECG and EDA signals, is determined by an overall synchrony and whether the same signals are reliable indicators of synchrony across individuals. As we have seen from the above discussed empirical example, this is not necessarily the case.

Thirdly, by applying the time-frequency based procedure from Muma et al. (2010), the proposed approach directly addresses problems of non-stationarity. In general, non-stationarity is a challenge in the examination of time series Scharf (1991); Vaseghi (2008). When a relationship between longitudinal data has to be quantified, non-stationarity leads to a underestimation of the time-dependent relationship (see examples from above).

Fourthly, a practical implication of the proposed approach is that researchers are given a simple two step tool, which is able to answer foundational research questions from emotion theory. The (real time) temporal resolution of synchrony of (non-stationary) measures allows for a person-orientated identification of response systems that have changing distributional characteristics over time (for example respiration rate). By addressing substantive questions on the intra-individual level the construct validity of concepts based on psychophysiological data can be strengthened (Borsboom et al., 2003).

Lastly, there are implications for emotion theory. The proposed approach facilitates assessing whether emotion eliciting stimuli lead to a stronger synchrony of psychophysiological reactions during positive and negative emotional states. In principle, within the time series representation of the state space model, treatment effects can be specified. With our empirical data collected from a study on humor we only found weak (descriptive) effects between two funny film clips. However, recent results from other studies on disgust indicate that differences between phases can be found. Based on our research, it is possible to conclude that testing for treatment effects in a time series is a precise and powerful tool which is in some cases superior to examining treatment effects with aggregated data.

### Limitations and future directions

The proposed approach provides novel possibilities for analyzing psychophysiological signals, nevertheless it also has some limitations. Both are briefly discussed: Although being able to incorporate non-stationary signals, the proposed approach is not capable of analyzing signals of constant amplitude. For example, when subjective data are measured using a potentiometer, participants can continuously adjust their subjective experience during emotional stimuli. Unfortunately, participants do not change their ratings continuously. Between changes in the position of the potentiometer, the signals are constant. For such time intervals, the proposed approach can not be used for the quantification of synchrony of physiological and subjective data. Nevertheless, participants have an enduring/varying emotional experience in this period. This general problem, which also occurs for (retrospective) paper-and-pencil ratings, remains unsolved. A potential solution could be an extension of the proposed approach, which operates on a feature space of the signals. Specifically, instead of looking directly at the bivariate coherence of measured signals, a feature estimation step could be added that extracts psychophysiological features from the signals (on a higher data level). The bivariate coherence from the first step may be based on the synchrony of these features. Enhanced robustness and a higher flexibility would possibly be the result. Also utilizing the feature space may save computational resources: Features change more slowly, which allows for reducing the sampling frequency in the coherence computation.

An additional limitation of the proposed approach is the assumed linearity of the bivariate coherences. In future work, the linear coupling between the signals could be relaxed, which would reflect a more realistic specification of underlying processes.

A possible future research direction consists in a detailed examination of coherence, and overall synchrony patterns, for specific individuals during specific emotion eliciting situations. On the one hand, individuals and their emotions could be recognized in applied settings (e.g., man-machine interaction). On the other hand, basic research questions of emotion theory could be addressed on a more detailed level. For this, further evaluation of more psychophysiological signal types in different emotion eliciting situations will be necessary. For example, the proposed approach works well in situations, in which emotions are elicited during a relatively long period of time (here several seconds). However, it is unclear how the proposed approach performs in the case of, for example, emotional priming, in which emotions are elicited for a very short period of time. The applicability of the proposed approach strongly depends on the responsiveness of physiological signals that were chosen for the analysis. Respiration, ECG, and EDA, for instance, are very slow or inadequate for the representation of affective responses in the context of priming. Therefore, adequate signals such as EEG waves should be chosen for the analysis.

From a practical perspective, it would be interesting to extend the use of the proposed approach to applications in the context of the strongly developing research field of affective computing (e.g., Picard, 1995, 1997; Scherer, 2010). Computers, which recognize, interpret, and process emotions could be used not only in health services, but also in education science, human-computer-interaction and other applications. Regarding the performance of the affect detection, the comparison of different algorithms (in terms of classifiers), would be an important field of research (Hudlicka, 2003; Kolodyazhniy et al., 2011), which also depends on the integration of different measured response signals, for example speech, physiological, and behavioral/mimic measures. Clearly, response signals differ in the sense that some are more informative than others, depending on which emotions need to be separated (e.g., Larsen et al., 2003).

Furthermore, as an important application, biofeedback might be considered (Thompson and Thompson, 2003). Since biofeedback addresses numerous signal types, such as behavior, muscle tone, brainwaves, breath, skin conductance, heart rate, temperature and pain perception, and many more, an adaptation of the proposed approach needs to be conducted in future research in order to be applicable in different settings (e.g., LaVaque, 2003; Dawson et al., 2007; Tassinary et al., 2007).

Finally, although beyond the scope of this article, an interesting field of research has emerged that examines the synchrony of psychophysiological signals and empathy (Marci and Orr, 2006; Hulsman et al., 2011; Oliveira-Silva and Gonçalves, 2011; Reed et al., 2013). In this research, couples of subjects have been examined with respect to their synchrony of psychophysiological responses in the context of empathy (Levenson and Ruef, 1992). Although, the neurobiological mechanisms underlying the synchronous empathic responses are mostly unclear, an adaption of the proposed approach might be helpful for further research in the field. The extension is not straightforward, because the inter-individual level is added to the current setting. Therefore, it might be interesting from a psychometric perspective to examine the synchrony of responses that stem from related but not identical subjects.

### Conflict of interest statement

The authors declare that the research was conducted in the absence of any commercial or financial relationships that could be construed as a potential conflict of interest.

## Footnotes

^1^In this paper a distinction between spectral coherence and synchrony is made. Spectral coherence is a well defined bivariate measure in the signal processing literature (Marple, 1987; Brillinger, 2001). The term “synchrony” defines an underlying latent state of an overall system-wise synchrony.

^2^On p. 143 of their publication, Hsieh et al. write: “There is a growing body of psychological literature questioning whether aggregates are useful descriptions of the individual (e.g., Nesselroade and Molenaar, 1999; Molenaar, 2004; Hamaker et al., 2005). Our ROC curve analyses were conducted at the individual level and this information was then compiled in all subsequent analytic steps. Although perhaps not an optimal approach, our analyses were built upon individual information.”
