# Supplementary material for: A new approach for the quantification of synchrony of multivariate non-stationary psychophysiological variables during emotion eliciting stimuli
Source: Front Psychol. 2015 Jan 20;5:1507. doi: 10.3389/fpsyg.2014.01507 (PMC4299432; doi:10.3389/fpsyg.2014.01507)
Supplement: Supplementary file 1 [file DataSheet1.ZIP › tutorial_code_bivariate_coherences.pdf]

Tutorial-style code for the extraction of the bi-variate coherences:

Contents

- Step 1: Split signals into segments and interpolate them to the same sampling frequency
- Step 2: Compute the bi-variate time-frequency coherences
- Step 3: Compute the time-frequency masks
- Step 4: Restrict the analysis to the six time-frequency coherence regions of interest
- Step 5: Compute average coherence within delineated regions for each time instant
- Function tfrCxy(x,y): Compute the bi-variate time-frequency coherence between two signals
- Function gauss2D(MM,sigma_t,sigma_f): Compute a Gaussian 2-D smoothing kernel
- Function mask(Xx): Compute a mask for EDA, respiration at the detected 3dB region.
- Function maskECG(x,Xx): Compute a mask for the ECG at the detected 3dB region.

Step 1: Split signals into segments and interpolate them to the same sampling frequency

```
% t_seg_eda_orig: the original sampling grid
% seg_eda_orig: the original eda_signal
% t_seg: the new common sampling grid
% A segment of an EDA signal
seg_eda = interp1(t_seg_eda_orig,seg_eda_orig,t_seg,'spline');
% A segment of a respiration signal
seg_re = interp1(t_seg_re_orig,seg_re_orig,t_seg,'spline');
% A segment of an ECG signal
seg_ecg = interp1(t_seg_ecg_orig,seg_ecg_orig,t_seg,'spline');
```

Step 2: Compute the bi-variate time-frequency coherences

```
% EDA and respiration
[C_eda_re,tfr_eda,tfr_re]=tfrCxy(seg_eda,seg_re);
% ECG and respiration
[C_ecg_re,tfr_ecg,tfr_re]=tfrCxy(seg_ecg,seg_re);
% EDA and ECG
[C_eda_ecg,tfr_eda,tfr_ecg]=tfrCxy(seg_eda,seg_ecg);
```

Step 3: Compute the time-frequency masks

```
Re_tfr_m = mask(tfr_re);
EDA_tfr_m = mask(tfr_eda);
ECG_tfr_m = maskECG(seg_ecg,tfr_ecg);
```

Step 4: Restrict the analysis to the six time-frequency coherence regions of interest

```
% Time-frequency coherence of EDA and respiration at respiration ...
    region
C_eda_re_m1=C_eda_re.*Re_tfr_m;
% Time-frequency coherence of EDA and respiration at eda region
C_eda_re_m2=C_eda_re.*EDA_tfr_m;
% Time-Frequency coherence of ECG and respiration at ECG region
C_ecg_re_m1=C_ecg_re.*ECG_tfr_m;
% Time-Frequency coherence of ECG and respiration at respiration ...
    region
C_ecg_re_m2=C_ecg_re.*Re_tfr_m;
% Time-Frequency coherence of EDA and ECG at EDA region
C_eda_ecg_m1=C_eda_ecg.*EDA_tfr_m;
% Time-Frequency coherence of EDA and ECG at ECG region
C_eda_ecg_m2=C_eda_ecg.*ECG_tfr_m;
```

Step 5: Compute average coherence within delineated regions for each time instant

```
for jj = 1:size(C_eda_re_m1)
    AvgCoh_eda_re_m1 = mean(C_eda_re_m1(C_eda_re_m1>0),1);
    AvgCoh_eda_re_m2 = mean(C_eda_re_m2(C_eda_re_m2>0),1);
    AvgCoh_ecg_re_m1 = mean(C_ecg_re_m1(C_ecg_re_m1>0),1);
    AvgCoh_ecg_re_m2 = mean(C_ecg_re_m2(C_ecg_re_m2>0),1);
    AvgCoh_eda_ecg_m1 = mean(C_eda_ecg_m1(C_eda_ecg_m1>0),1);
    AvgCoh_eda_ecg_m2 = mean(C_eda_ecg_m2(C_eda_ecg_m2>0),1);
end
```

Function tfrCxy(x,y): Compute the bi-variate time-frequency coherence between two signals

```
function [Cxy,Xx,Yy] = tfrCxy(x,y)
% inputs:
%            - x: a given segment of physiological signal 1
%            - y: a given segment of physiolocical signal 2
% outputs:
```

```
%            - Cxy: time-frequency coherence between x and y
%            - Xx: time-frequency representation of x
%            - Yy: time-frequency representation of y

% Compute the spectrogram, e.g., using the Time-Frequency ... Toolbox (TFTB)
% that is istributed under the terms of the GNU Public Licence, and
% available at http://tftb.nongnu.org
N = length(x);
Xx = tfrsp(x,1:N,N,hamming(Nw),0); % e.g., use a window of ... length Nw=N/4
Yy = tfrsp(y,1:N,N,hamming(Nw),0);

% Compute a 2-D Gaussian smoothing kernel of size (MM x MM) with
% parameters sigma_t, sigma_f
h = gauss2D(MM, sigma_t, sigma_f); %

% Smooth the time-frequency distributions using the 2-D Gaussian ... kernel
Pxx = Xx2.^2; Pxxh = conv2(Pxx,h);
Pyy = Yy2.^2; Pyyh = conv2(Pyy,h);
Pxy = Yy2.*conj(Xx2); Pxyh = conv2(Pxy,h);

% Compute the time-frequency coherence (crop by to the size of
% time-frequency distributions, since the convolution increased ... the size by
% MM
Cxy = abs(Pxyh(MM/2:end-MM/2,MM/2:end-MM/2))...
./((Pxxh(MM/2:end-MM/2,MM/2:end-MM/2)...
.*Pyyh(MM/2:end-MM/2,MM/2:end-MM/2))).^0.5;
end
```

Function gauss2D(MM,sigma_t,sigma_f): Compute a Gaussian 2-D smoothing kernel

```
function [h] = gauss2D(MM,sigma_t,sigma_f)
% inputs:
%            - MM: size of the kernel is (MM x MM)
%            - sigma_t, sigma_f: standard deviations in time and ... frequency
%            of the 2-D Gaussian function
% output:
%            - h: Gaussian 2-D kernel
     [x,y] = meshgrid(-MM:MM,-MM:MM);
     arg   = -(((x.*x)/sigma_t+(y.*y)/sigma_f)/2);
     h     = exp(arg);
     h(h<eps*max(h(:))) = 0;
     sumh = sum(h(:));
     if sumh ≠ 0,
       h  = h/sumh; % normalize the kernel
     end;
end
```

Function mask(Xx): Compute a mask for EDA, respiration at the detected 3dB region.

```
function [Xx_m] = mask(Xx)
% inputs:
%           Xx: a time-frequency signal
% output:
%           Xx_m: the mask
Band=[];
Xx_m=zeros(size(Xx));
% finding the 3 dB region
    for jj=1:size(Xx,2)
        % Xx(:,jj): "local spectrum" at time instance jj
        ind_f0_low=find(Xx(:,jj)/max(Xx(:,jj))>.5,1,'first');
        [resp_max,ind_f0m]=max(Xx(:,jj));
        ind_f1=find(Xx(:,jj)/max(Xx(:,jj))<0.5,100,'first');
        idx1=find(ind_f1>ind_f0m,1,'first');
        ind_f0_high=ind_f1(idx1);
        Band=[Band; {ind_f0_low:ind_f0_high}];
        % the mask takes value 1 at signal's 3 dB region and ...
            zero, else
        Xx_m(Band{jj,1},jj)=1;
    end
end
```

Function maskECG(x,Xx): Compute a mask for the ECG at the detected 3dB region.

```
function [ECG_tfr_m] = maskECG(x,Xx)
% inputs:
%           seg_ecg: a time-domain ECG signal
%           Xx: corresonding time-frequency ECG signal
% output:
%           Xx_m: the mask
B0ecg=[];
ECG_tfr_m=zeros(size(Xx));

Fs=100;
NFFT = 2^nextpow2(length(x)); % Next power of 2 from length of x
ECG_spec = abs(fft(x,NFFT)).^2;
f_ECG=linspace(0,50,length(x));
f_FFT = Fs/2*linspace(0,1,NFFT/2);
f_FFT_uTh=find(f_FFT>2,1,'first');
f_FFT_lTh=find(f_FFT<.6,1,'last');
[max_FFT, ind_max_FFT]=max(ECG_spec(f_FFT_lTh:f_FFT_uTh));
f0_fft_est=f_FFT(f_FFT_lTh+ind_max_FFT); % rough estimate of ...
    pulse freq.
ind_f0_u=find(f_ECG>f0_fft_est+.3,1,'first');
ind_f0_l=find(f_ECG<f0_fft_est-.3,1,'last');
Δ_01=find(f_ECG>.1,1,'first');
```

```
    % finding the mask, given a rough estimate of the pulse ...
        frequency
    for jj=1:size(Xx,2)
        % "local spectrum" at time instance jj
        ECG_psd=tfr_eda(:,jj);
        % first rough estimate of the band around the maximum
        [pulse_max,ind_f0m]=max(ECG_psd(ind_f0_l:ind_f0_u));
        ind_f0m=ind_f0_l+ind_f0m;
        pulse_3dB=.5*pulse_max;
        f0_low_temp= f_ECG(ind_f0m)-.4;
        f0_high_temp=f_ECG(ind_f0m)+.4;
        ind_f0_low_temp=find(f_ECG>f0_low_temp,1,'first');
        ind_f0_high_temp=find(f_ECG<f0_high_temp,1,'last');
        ind_f0_low=ind_f0_low_temp+find(ECG_psd(ind_f0_low_temp:ind_f0m)...
                                                              <pulse_3dB,1,'last');
        if isempty(ind_f0_low)==1
            ind_f0_low=ind_f0m-Δ_01;
        end
        % estimates based on 3dB level
        ind_f0_high=ind_f0m+find(ECG_psd(ind_f0m:ind_f0_high_temp)...
                                                             <pulse_3dB,1,'first');
        if isempty(ind_f0_high)==1
            ind_f0_high=ind_f0m+Δ_01;
        end
        B0ecg=[B0ecg; {ind_f0_low:ind_f0_high}];
        % ECG mask takes value 1 at pulse delineated region and ...
            zero, else
        ECG_tfr_m(B0ecg{jj,1},jj)=1;
    end
```
